# The SPI-2 type III secretion system restricts motility of *Salmonella*-containing vacuoles

**DOI:** 10.1111/j.1462-5822.2007.00977.x

**Published:** 2007-06-07

**Authors:** Amy E Ramsden, Luís J Mota, Sylvia Münter, Spencer L Shorte, David W Holden

**Affiliations:** 1 Centre for Molecular Microbiology and Infection, Imperial College London Armstrong Road, London SW7 2AZ, UK.; 2 Plate-Forme d'Imagerie Dynamique (PFID), Département de Biologie, Cellulaire et Infection, Institut Pasteur 25, Rue du Docteur Roux, 75724 Paris Cedex 15, France

## Abstract

Intracellular replication of *Salmonella enterica* occurs in membrane-bound compartments, called *Salmonella*-containing vacuoles (SCVs). Following invasion of epithelial cells, most SCVs migrate to a perinuclear region and replicate in close association with the Golgi network. The association of SCVs with the Golgi is dependent on the *Salmonella-*pathogenicity island-2 (SPI-2) type III secretion system (T3SS) effectors SseG, SseF and SifA. However, little is known about the dynamics of SCV movement. Here, we show that in epithelial cells, 2 h were required for migration of the majority of SCVs to within 5 μm from the microtubule organizing centre (MTOC), which is located in the same subcellular region as the Golgi network. This initial SCV migration was saltatory, bidirectional and microtubule-dependent. An intact Golgi, SseG and SPI-2 T3SS were dispensable for SCV migration to the MTOC, but were essential for maintenance of SCVs in that region. Live-cell imaging between 4 and 8 h post invasion revealed that the majority of wild-type SCVs displaced less than 2 μm in 20 min from their initial starting positions. In contrast, between 6 and 8 h post invasion the majority of vacuoles containing *sseG*, *sseF* or *ssaV* mutant bacteria displaced more than 2 μm in 20 min from their initial starting positions, with some undergoing large and dramatic movements. Further analysis of the movement of SCVs revealed that large displacements were a result of increased SCV speed rather than a change in their directionality, and that SseG influences SCV motility by restricting vacuole speed within the MTOC/Golgi region. SseG might function by tethering SCVs to Golgi-associated molecules, or by controlling microtubule motors, for example by inhibiting kinesin recruitment or promoting dynein recruitment.

## Introduction

*Salmonella enterica* serovar Typhimurium (*S.* Typhimurium) is a facultative intracellular pathogen that causes a self-limiting gastroenteritis in humans and a typhoid-like illness in susceptible mouse strains. Intracellular replication of this bacterium occurs in a specialized membrane-bound compartment known as the *Salmonella*-containing vacuole (SCV). The pathogenesis of *S*. Typhimurium involves two type III secretion systems (T3SSs), which deliver effector proteins across host cell membranes. The *Salmonella*-pathogenicity island 1 (SPI-1)-encoded T3SS is expressed by extracellular bacteria and its effector proteins, which are delivered through the plasma membrane, trigger bacterial invasion and influence the early steps of SCV biogenesis (Schlumberger and Hardt, 2005). The SPI-2-encoded T3SS is activated by intracellular bacteria and is required for systemic virulence in the murine model of salmonellosis (Shea *et al*., 1996; Cirillo *et al*., 1998). Effectors translocated by the SPI-2 T3SS into and across the SCV membrane facilitate intracellular multiplication of *S*. Typhimurium in different cells types, including macrophages and epithelial cells (Abrahams and Hensel, 2006).

SseG, SseF and SifA are SPI-2 T3SS effectors that are necessary for the formation in epithelial cells of tightly clustered bacterial microcolonies in close proximity to the Golgi apparatus (Salcedo and Holden, 2003; Boucrot *et al*., 2005; Abrahams *et al*., 2006; Deiwick *et al*., 2006). In uninfected epithelial cells, the Golgi apparatus is a compact structure, composed of dozens of connected stacks of cisternae that are localized close to the microtubule organizing centre (MTOC) and the nuclear membrane (Farquhar and Palade, 1981). However, in infected cells the morphology of the Golgi network is often distorted by the *S.* Typhimurium microcolony (Salcedo and Holden, 2003). Different lines of evidence suggest that the spatial localization of SCVs and *S.* Typhimurium – Golgi interactions are important for bacterial replication. First, SseG and SseF are both required for intracellular multiplication in macrophages and epithelial cells (Hensel *et al*., 1998; Salcedo and Holden, 2003; Abrahams *et al*., 2006; Deiwick *et al*., 2006). Second, in epithelial cells, more bacterial replication occurs if SCVs are in the vicinity of the Golgi network (Salcedo and Holden, 2003). Third, dissolution of the Golgi apparatus with brefeldin A (BFA) inhibits the intracellular growth of *S.* Typhimurium (Salcedo and Holden, 2003). Furthermore, in infected cells, exocytic transport vesicles transiting from the Golgi to the plasma membrane are redirected and accumulate near SCVs in an SseG- and SseF-dependent manner (Kuhle *et al*., 2006). However, no direct fusion between exocytic vesicles and SCVs has been observed and the physiological significance of the physical proximity between *S*. Typhimurium microcolonies and the Golgi network remains unclear.

Most studies addressing the spatial localization of SCVs have relied on the analysis of chemically fixed infected cells by immunofluorescence microscopy. However, SCV positioning is determined by the properties of vacuole movement, which can only be analysed by live-cell microscopy. In this work, we undertook a detailed study of SCV positioning (in fixed cells) and movement (in living cells) at different times after invasion of epithelial cells. We investigated the contribution of microtubules, the Golgi network and the SPI-2 T3SS. Our results indicate that that SseG and SseF control SCV positioning by restricting their microtubule-dependent motility. The action of SseG appears to be localized to the Golgi region and SCV positioning is also dependent on an intact Golgi network, suggesting that an SseG/SseF complex could function by tethering SCVs to Golgi-related molecules.

## Results

### Effect of nocodazole and BFA on the migration of SCVs to the MTOC/Golgi region within host cells

**Fig. 1:**
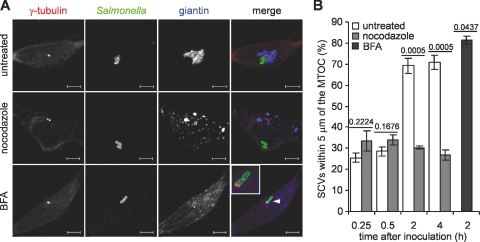
Migration of SCVs to the MTOC/Golgi region of HeLa cells. A. Representative images of HeLa cells infected with wt *S.* Typhimurium for 2 h, in the presence or absence of nocodazole or BFA. Cells were fixed in ice-cold methanol and coimmunolabelled for giantin (blue in merged images), γ-tubulin (red in merged images) and *S.* Typhimurium (green in merged images). Images were acquired by confocal microscopy and represent combined projections of multiple Z-sections. Higher magnification of cells in boxed region. Scale bars, 5 μm. B. Percentage of SCVs within 5 μm of the MTOC in BFA-, nocodazole-treated or untreated HeLa cells. *P*-values are indicated above bars and were obtained by comparing nocodazole- or BFA-treated cells to untreated cells at the same time point.

In previous work it was shown that by 8 h post invasion (p.i.) in epithelial cells, the majority of vacuoles enclosing wild-type (wt) *S.* Typhimurium were in close proximity to the Golgi apparatus (Salcedo and Holden, 2003). Within epithelial cells, the MTOC and the Golgi apparatus are positioned close to each other on one side of the nucleus (Tassin *et al*., 1985; Kronebusch and Singer, 1987). To examine the relationship between centripetal migration of SCVs and the Golgi apparatus and the MTOC in more detail, HeLa cells were either untreated or exposed to nocodazole or BFA and fixed at 0.25 h, 0.5 h, 2 h and 4 h after invasion by wt bacteria. Cells were then labelled for the MTOC (anti-γ-tubulin antibody), the Golgi apparatus (anti-giantin antibody) and *S.* Typhimurium (anti-*Salmonella* antibody). At 0.25 h and 0.5 h p.i. of untreated cells, approximately 30% of SCVs were found within 5 μm of the MTOC; however, at 2 h and 4 h p.i. approximately 70% of SCVs were present in this region of the cell (Fig. 1).

At 0.25 h and 0.5 h after invasion of nocodazole-treated cells, the percentage of SCVs within 5 μm of the MTOC was similar to that in untreated cells (Fig. 1B). However, nocodazole treatment caused a dramatic reduction in the percentage of SCVs within 5 μm of the MTOC at 2 h and 4 h p.i. (Fig. 1). Disruption of microtubules by nocodazole causes Golgi stacks to disconnect from each other, redistributing from their normal perinuclear location to endoplasmic reticulum exit sites (Fig. 1A) (Cole *et al*., 1996). To examine the influence of the Golgi apparatus in the centripetal migration of SCVs, infected cells were treated with BFA at 15 min p.i. and fixed 1 h 45 min later. BFA treatment results in fragmentation of the Golgi and redistribution of its components into the endoplasmic reticulum (Lippincott-Schwartz *et al*., 1989). BFA treatment caused an increase in the proportion of SCVs within 5 μm of the MTOC (Fig. 1B). Indeed, in BFA-treated but not untreated cells, bacteria were occasionally observed to colocalize with γ-tubulin (Fig. 1A). These results indicate that microtubules are required for centripetal migration of SCVs, and that the Golgi network might obstruct migration of SCVs towards the minus ends of microtubules.

### Analysis of SCV motility at early stages of infection

**Fig. 2:**
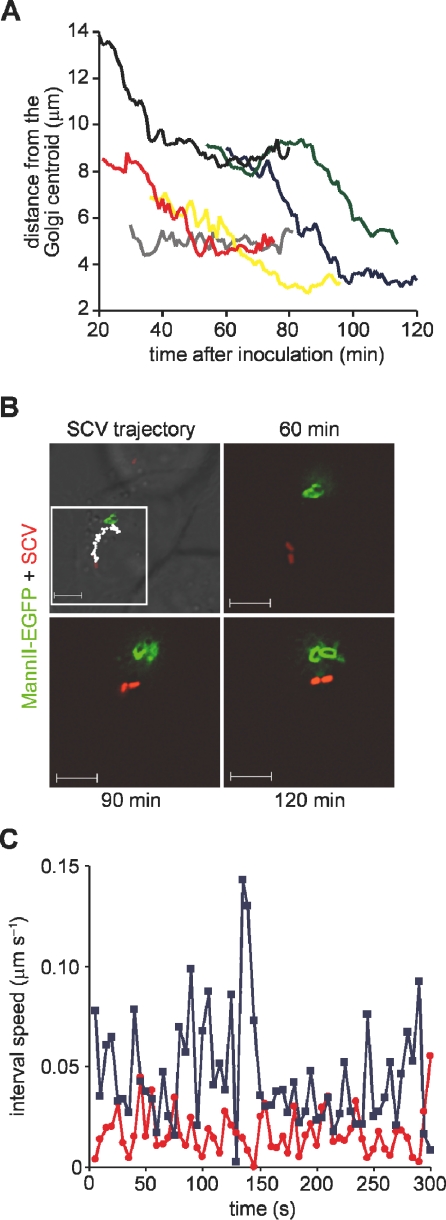
Time-lapse video microscopy analysis of the movement of SCVs towards the Golgi in HeLa cells. To allow visualization of the Golgi, HeLa cells were transiently transfected with a vector encoding MannII-EGFP. Transfected cells were infected with wt *S.* Typhimurium expressing the red fluorescent protein (DsRed). A. Graphical representation of the distance between red fluorescence centroid (*Salmonella*) and green fluorescence centroid (Golgi) in every frame of six time-lapse sequences; images were taken at 1 min intervals. Each colour represents a single SCV. B. In the upper left panel an SCV trajectory (corresponding to the blue curve in Fig. 2A), is superimposed on the fluorescent and DIC image of the infected HeLa cell. The boxed region is magnified in subsequent panels, which show merged fluorescent images at 30 min intervals (see Supplementary Movie S1). Scale bar, 5 μm. C. Change of speed over time is shown for two distinct SCVs. Data are from time-lapse sequences where images were acquired at 5 s intervals. One SCV (blue line), had an average speed of 0.045 ± 0.004 μm; the other (red line) had an average speed of 0.017 ± 0.001 μm s^−1^.

To further characterize the migration of SCVs towards the MTOC-Golgi region, we analysed the movement of individual SCVs by time-lapse fluorescence microscopy. As a marker for the Golgi network HeLa cells were transfected with a plasmid encoding a chimeric protein comprising murine Mannosidase II (MannII) fused at its C-terminus to enhanced green fluorescent protein (EGFP). MannII is a Golgi-resident protein, mainly localized at the medial portion of the stack (Novikoff *et al*., 1983). HeLa cells transfected for approximately 20 h with the MannII-EGFP vector were infected with wt *S.* Typhimurium 12023 harbouring the pDsRed plasmid. We then analysed the movement of 10 different SCVs by acquiring images over 50–60 min periods at 1 min intervals between 20 min and 120 min p.i. To determine SCV migration relative to the Golgi region, the positions of the centroids corresponding to DsRed and MannII-EGFP fluorescence were calculated in each image of every time-lapse sequence. The movement of six representative SCVs in relation to the MannII-EGFP centroid are shown graphically in Fig. 2A, and a typical example of SCV migration is shown in the time-lapse panels of Fig. 2B, and in Supplementary Movie S1. SCV movements were saltatory, and characterized by frequent changes in direction relative to the MannII-EGFP centroid. Analysis of the initial and final distance of the 10 SCVs showed that in 1 h they displaced on average 3.5 ± 0.9 μm towards the MannII-EGFP centroid.

*Salmonella*-containing vacuole migration towards the Golgi could be explained by a greater number of centripetal relative to centrifugal displacements, different speeds depending on direction, or a combination of the two. Therefore, we determined the directionality and speed of SCVs between 20 min and 120 min p.i. For these experiments we analysed time-lapse sequences over 5 min periods at 5 s intervals. A total of 52 randomly selected SCVs were imaged, corresponding to 3118 distinct 5 s displacements. There were large differences in SCV speed, not only between distinct SCVs, but also between the different displacements of a single SCV (Fig. 2C). The mean speed per SCV was 0.024 ± 0.002 μm s^−1^, with a maximum of 0.064 μm s^−1^ and a minimum of 0.007 μm s^−1^. To analyse the directionality of SCV trajectories, each 5 min trajectory and its component 5 s displacements were classified as centripetal if it brought the SCV closer to the MannII-EGFP centroid, or centrifugal, if it moved further away. While there was no significant difference (*P* = 0.13) between the average speeds of SCVs moving centripetally (0.026 ± 0.002 μm s^−1^, *n* = 27) and centrifugally (0.022 ± 0.002 μm s^−1^, *n* = 25), the average number of centripetal displacements per SCV (30.8 ± 0.6) was significantly higher (*P* = 0.035) than that of centrifugal displacements per SCV (29.1 ± 0.9). These results indicate that SCV movement towards the Golgi is caused mainly by a greater number of centripetal relative to centrifugal displacements, rather than greater speed of SCVs moving in a centripetal direction.

### Requirement of the SPI-2 T3S to retain SCVs at the MTOC/Golgi region

Previous work has shown that the *S.* Typhimurium SPI-2 T3SS and the effector SseG are required for bacterial association with the Golgi apparatus between 6 and 8 h p.i. (Salcedo and Holden, 2003). To examine the distances between vacuoles containing SPI-2 mutant bacteria and the MTOC at these and earlier time-points, HeLa cells were infected with wt bacteria or strains carrying mutations in either *ssaV*[which encodes an essential component of the SPI-2 T3SS machinery (Deiwick *et al*., 1998)] or *sseG*. Infected cells were fixed at 2 h intervals from 2 h to 8 h and examined by confocal microscopy.

**Fig. 3:**
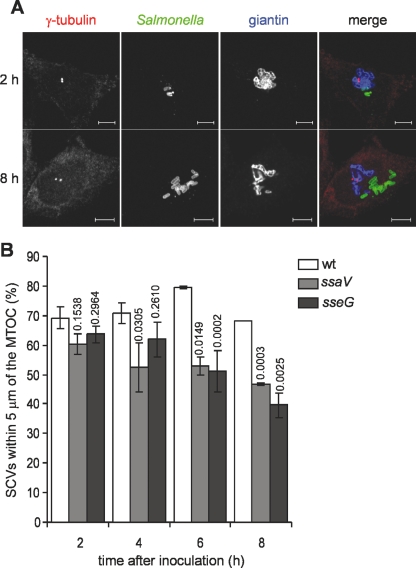
Requirement of the SPI-2 T3SS and effector SseG for SCV association with the MTOC. A. Representative images of HeLa cells infected for 2 h and 8 h with *sseG* mutant *S.* Typhimurium. Infected cells were fixed in ice-cold methanol and triple labelled for *Salmonella* (green in merged images), giantin (blue in merged images) and γ-tubulin (red in merged images). Cells were analysed by confocal microscopy and images represent combined projections of multiple z-sections. Scale bars, 5 μm. B. Percentage of *ssaV* mutant, *sseG* mutant and wt *S.* Typhimurium bacteria within 5 μm from the MTOC over an 8 h time-course. Error bars not apparent if less than 0.1; *P*-values are indicated above bars, compared with corresponding wt values.

At 2 h and 4 h after infection, only minor differences were observed in the number of bacteria within 5 μm of the MTOC between cells infected with wt, *ssaV* or *sseG* mutant bacteria (Fig. 3). However, at 6 h and 8 h after infection, lower numbers of vacuoles containing *ssaV* or *sseG* mutant bacteria in comparison to vacuoles containing wt bacteria were observed at distances within 5 μm from the MTOC (Fig. 3). At 8 h p.i. more than 50% of *ssaV* or *sseG* mutant bacteria were at a distance greater than 5 μm from the MTOC. This indicates that SPI-2 encoded T3SS and SseG are not involved in SCV migration to the MTOC but are required for continued association of SCVs with this region of the cell.

### Requirement of an intact Golgi apparatus for SCV association with the MTOC

**Fig. 4:**
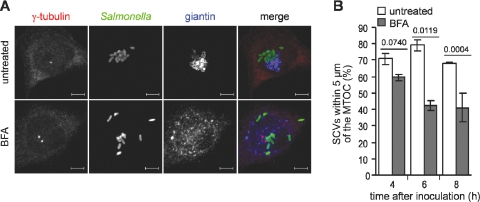
Effect of BFA on SCV association with the MTOC. A. HeLa cells were infected with wt *S.* Typhimurium and then incubated in the presence or absence of BFA. Cells were then fixed at 8 h post inoculation and coimmunolabelled for γ-tubulin (red in merged images), *Salmonella* (green in merged images) and giantin (blue in merged images) and analysed by confocal microscopy. Images represent combined projections of multiple z-slices. Scale bars, 5 μm. B. Percentage of bacteria within 5 μm of the MTOC at 4 h, 6 h and 8 h p.i. *P*-values are indicated above bars and are derived from comparisons with corresponding untreated cells.

We have previously shown that when expressed ectopically, SseG localizes to the Golgi apparatus and can rescue the Golgi association defect of an *sseG* mutant strain (Salcedo and Holden, 2003). We therefore examined the requirement for an intact Golgi apparatus for the association between SCVs and the MTOC. To this end HeLa cells infected with wt *S.* Typhimurium were treated with BFA at 15 min p.i and then fixed at 4 h, 6 h and 8 h p.i. At 4 h p.i. the percentage of SCVs within 5 μm of the MTOC was slightly lower (60 ± 3%) than in untreated cells (71 ± 3%), and there was a significant reduction in the numbers of SCVs within 5 μm of the MTOC in BFA-treated cells at both 6 h and 8 h p.i. (Fig. 4). However, BFA treatment had no noticeable effect on the distribution of vacuoles containing *sseG* mutant bacteria at 8 h p.i. (data not shown). This indicates that although not required for migration of SCVs to the MTOC, the Golgi seems to have an important role in the retention of SCVs in this region of the cell.

### Requirement of an intact microtubule network for the redistribution of *sseG* mutant *S.* Typhimurium away from the MTOC/Golgi region

**Fig. 5:**
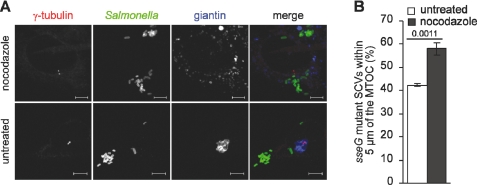
Effect of nocodazole on the redistribution of vacuoles containing *sseG* mutant bacteria. A. Representative images of HeLa cells infected with wt *S.* Typhimurium, either left untreated or exposed to nocodazole at 2 h p.i., and fixed 6 h later. Cells were labelled for *Salmonella* (green in merged images), giantin (blue in merged images) and γ-tubulin (red in merged images) and analysed by confocal microscopy. Images represent combined projections of multiple z-slices; scale bars correspond to 5 μm. B. Percentage of *sseG* mutant bacteria within 5 μm of the MTOC following addition of nocodazole; *P*-value is indicated above bars.

To determine whether microtubules are required for the redistribution of vacuoles containing *sseG* mutant bacteria following their initial migration to the MTOC/Golgi region, HeLa cells infected with the *sseG* mutant were treated with nocodazole at 2 h p.i. to depolymerize microtubules and fixed 6 h later for microscopy analysis. In untreated cells, 42 ± 3% of *sseG* bacteria were within 5 μm of the MTOC (Fig. 5), similar to that observed previously (Fig. 3B). In nocodazole-treated cells the percentage of mutant bacteria observed within 5 μm of the MTOC was significantly higher and similar to that observed for *sseG* mutant SCVs in untreated cells at 2 h p.i. (Fig. 3B). This suggests that the centrifugal redistribution of SCVs by 8 h p.i. involves movement on microtubules.

### Analysis of SCV motility at late stages of infection

To characterize the dynamics of SCVs containing wt, *ssaV* or *sseG* mutant bacteria following their migration to the MTOC/Golgi region, we examined their motility in relation to the Golgi network by time-lapse fluorescence microscopy between 4 h and 8 h p.i. Preliminary experiments suggested that while the motility of wt SCVs was limited, many vacuoles containing *ssaV* and *sseG* mutant bacteria were capable of rapid and apparently erratic movement in infected cells. To quantify this phenotype, the positions of centroids of SCVs were determined in time-lapse sequences where images were acquired every min over a 20 min time-course. SCVs were analysed in two time periods: between 4 h and 6 h p.i. (20 wt SCVs, 21 *ssaV* SCVs and 29 *sseG* SCVs), and between 6 h and 8 h p.i. (30 wt SCVs, 22 *ssaV* SCVs and 23 *sseG* SCVs). SCVs were then classified as undergoing large or small displacements depending on whether their maximum displacement from their initial position exceeded, or was less than 2 μm, over the 20 min time-course.

**Fig. 6:**
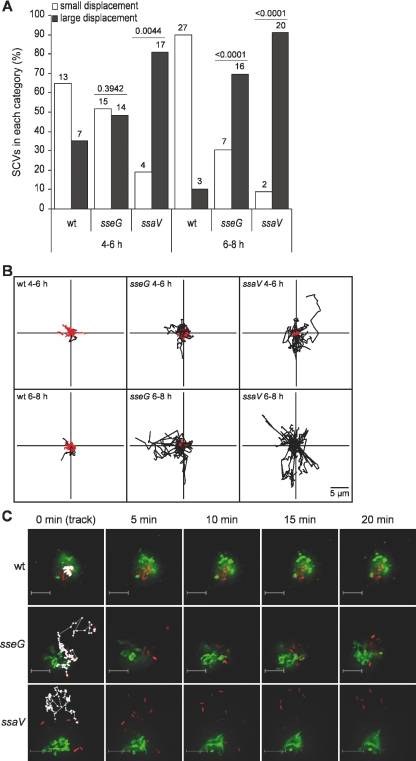
Live imaging analysis of wt, *ssaV* and *sseG* mutant SCVs. A. Percentages of small (less than 2 μm) and large (greater than 2 μm) displacing wt, *ssaV* and *sseG* mutant SCVs between 4 h and 6 h p.i., and between 6 h and 8 h p.i. The number of SCVs for each category is indicated above the bar, as is the *P*-value from comparing *sseG* and *ssaV* mutant SCVs with wt SCVs within the same time period. B. Trajectories of all vacuoles sampled, containing wt, *ssaV* and *sseG* mutant bacteria. SCV trajectories were reoriented to start at *x,y* = 0 in a standardized grid; small displacing SCVs are in red and large displacing SCVs are in black. C. Images from *Supplementary material* Movies S2–4. ManII-EGFP-expressing HeLa cells, infected with wt (upper panel), *sseG* mutant (middle panel) or *ssaV* mutant (lower panel) DsRed-expressing *S.* Typhimurium. The first image shows full SCV trajectories superimposed on the merged image at 0 min; the consecutive images are 5 min apart. Scale bar, 5 μm. Images were acquired every min for 20 min.

Between 4 h and 6 h p.i., the majority of wt SCVs underwent small displacements, whereas the majority of *ssaV* SCVs showed large displacements; there was a nearly equivalent distribution of *sseG* vacuoles in these categories (Fig. 6A and B). Between 6 h and 8 h p.i., 70% of *sseG* SCVs and 90% of *ssaV* SCVs underwent major displacements, compared with only 10% of wt SCVs (Fig. 6A and B). Examples of the movement of individual wt, *ssaV* and *sseG* SCVs are shown in the time-lapse sequences of Fig. 6C and in Supplementary Movies S2 and S3. Trajectories of small (red lines) or large (black lines) displacements of wt, *ssaV* and *sseG* SCVs are shown in Fig. 6B. Trajectories were orientated so that their initial displacement vectors within a standardized grid started at x,*y* = 0 (Lacayo and Theriot, 2004). Among the vacuoles undergoing large displacements, several containing *ssaV* and *sseG* mutant bacteria displayed dramatic displacement over relatively large distances (Fig. 6B): between 6 and 8 h the maximum displacement observed for an *sseG* vacuole was 13.6 μm and 10 SCVs displaced further than 5 μm (mean displacement = 4.24 ± 0.76 μm, *n* = 23). Similar results were observed for *ssaV* SCVs where the maximum displacement observed was 10.3 μm with 10 SCVs displacing further than 5 μm (mean displacement = 4.97 ± 0.56 μm, *n* = 22). By contrast the maximum displacement observed for a wt vacuole was 4.85 μm (mean displacement = 1.49 ± 0.15 μm, *n* = 30).

Vacuoles containing *sseF* or *sseFG* double mutant bacteria that were imaged between 4 and 8 h p.i. showed an almost indistinguishable phenotype to that observed with *sseG* (Supplementary Fig. S1). The greater number of *ssaV*, *sseG*, *sseF* and *sseFG* SCVs showing large displacements relative to wt SCVs could be due to differences in directionality, speed, or a combination of both. To determine SCV directionality and speed, wt, *sseG* and *ssaV* vacuoles were imaged every 5 s over 5 min periods between 6 h and 8 h p.i. SCVs were then grouped as showing large or small displacements. Because we were interested in analysing the speed and directionality of the less frequently observed wt SCVs undergoing large displacements and *ssaV* and *sseG* SCVs showing small displacements, infected cells were intentionally sampled to obtain a representative number of these more uncommon events.

**Fig. 7:**
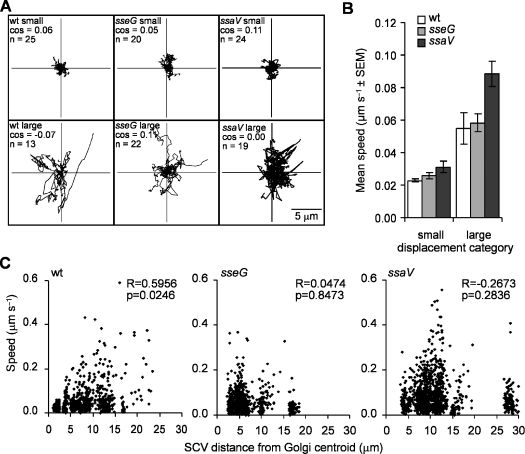
Analysis of wt, *ssaV* and *sseG* vacuole directionality and speed between 6 h and 8 h p.i. SCVs were imaged every 5 s for 5 min. A. Trajectories of small and large displacing vacuoles containing wt, *ssaV* and *sseG* mutant bacteria. Trajectories were reoriented to start at *x,y* = 0 with the first displacement vector pointing in the same direction (+x, +y) in a standardized grid. The number of SCVs examined and the average cosine are indicated for each category. B. Mean speeds of small and large displacing vacuoles containing wt, *ssaV* and *sseG* bacteria. C. Scatter plot in which the speeds of individual 5 s movements of vacuoles containing wt, *sseG* and *ssaV* bacteria undergoing large displacements were plotted against the distance of the vacuole from the Golgi centroid at the end of its 5 s movement. Spearman correlation *R*-value and *P*-values are indicated.

As for the experiments shown in Fig. 6, the direction of SCV movement was first examined by tracing trajectories of SCVs having large or small displacements, after they had been reorientated so that their initial displacement vectors within a normalized grid started at x,*y* = 0, positive for *x* and *y* in the first vector. Visual inspection of trajectories revealed that irrespective of genotype, SCVs undergoing large displacements moved within a 15 μm radius (Fig. 7A). Furthermore, regardless of the extent of displacement or bacterial genotype, movement occurred in all quadrants of the grid (Fig. 7A). This indicates that there was no significant impact of SCV directionality on their motility. To analyse this further, we quantified the directionality of SCV motility by calculating the cosine of the internal angle between each pair of consecutive displacement vectors. Regardless of the bacterial genotype, the differences between the mean cosine values of SCVs having large or small displacements were minimal (Fig. 7A). Furthermore, the mean cosine values were close to zero, which confirms that the SCVs moved randomly regardless of genotype and motility.

Analysis of SCV speeds revealed that, irrespective of bacterial genotype, SCVs undergoing large displacements moved faster than those undergoing small displacements, and that the small number of wt SCVs classified as having large displacements travelled at similar speeds to *sseG* SCVs in the same category (Fig. 7B). Vacuoles containing *ssaV* mutant bacteria that showed large displacements moved on average at faster speeds (Fig. 7B). Therefore, SCVs that showed large displacements did so because they moved faster, rather than exhibiting altered directionality. This implies that the SPI-2 T3SS system and SseG influence SCV motility by restricting vacuole speed.

We next investigated SCV speed in relation to the Golgi network. Each 5 s SCV displacement was classified according to whether it brought the vacuole closer to the MannII-EGFP centroid or further away. As for the early stage of infection, there were no significant differences between the speeds of vacuoles containing wt, *sseG* or *ssaV* mutant bacteria moving in centripetal and centrifugal directions (data not shown). A scatter plot was made between the speed of each SCV and the distance from the Golgi centroid at the end of each 5 s imaging period. No relationship between speed and distance from the Golgi was observed for SCVs undergoing small displacements (data not shown). However, a correlation (*R* = 0.596) was obtained with wt SCVs displaying large displacements (Fig. 7C). This indicates that motility of wt SCVs is restricted when they are within close proximity to the Golgi, but not when they are further away. If SseG only functions close to the Golgi network, distance from the Golgi should have no effect on the speed of *sseG* mutant SCVs and indeed this is what was observed (Fig. 7C). This result indicates that SseG acts by restricting the motility of SCVs that are within the proximity of the Golgi, but that it does not limit the motility of SCVs that are further away.

## Discussion

During the first h following invasion of epithelial cells by *S.* Typhimurium, the majority of SCVs mature into a compartment that has some characteristics of late endosomes but which does not interact with lysosomes (Meresse *et al*., 1999a). During this period, SCVs migrate centripetally from the cell periphery to a perinuclear location. Perinuclear migration involves the recruitment of active Rab7, its effector RILP, the dynein motor (with which RILP is known to interact) and an intact microtubule network (Meresse *et al*., 1999b; Guignot *et al*., 2004; Harrison *et al*., 2004; Marsman *et al*., 2004). This suggests that like chlamydial inclusions (Grieshaber *et al*., 2003), nascent SCVs engage the dynein motor and undergo minus-end directed movement towards the MTOC. In the present study we have used live microscopy of infected cells to show that movement of these early SCVs is saltatory, and that they undergo frequent changes in direction. Net centripetal migration is caused by a greater number of centripetal to centrifugal movements, rather than increased speed of SCVs when moving in a centripetal direction. Such bidirectional movements are typical of many organelles (Vale *et al*., 1992) and probably reflect an alternating cycle of recruitment or activation of plus-end directed kinesin, and minus-end directed dynein motors on the SCV. Hence, net centripetal migration of SCVs to the MTOC/Golgi region would occur if the overall balance of active motors on the SCV favours dynein. It is also possible that dynamic instability of microtubules might contribute to SCV movement, as in the case of phagosome movement in macrophages (Blocker *et al*., 1998). Relatively little is known about the intracellular motility of bacterial pathogens that are enclosed by a vacuolar membrane. *Chlamydia trachomatis* migrates to the MTOC at a speed of approximately 0.1 μm s^−1^ (Grieshaber *et al*., 2003). SCVs are considerably larger than *Chlamydia* inclusions, and the average speeds during this stage of infection are considerably lower (approximately 0.02 μm s^−1^).

Previous work from our laboratory showed that by 8 h p.i., the majority of SCVs form a microcolony that is closely associated with the Golgi apparatus. In HeLa cells the Golgi comprises a compact array of stacks positioned close to the MTOC on one side of the nucleus. In this work we observed that exposure of cells to BFA shortly after infection resulted in a greater number of vacuoles containing wt bacteria within 5 μm of the MTOC. This is consistent with the observation that in infected HeLa cells, the morphology of the Golgi apparatus is frequently distorted (Salcedo and Holden, 2003). Both observations suggest that SCVs and Golgi membranes compete for a position close to the MTOC and that replicating bacteria physically occlude Golgi membranes from occupying their normal position in the cell. The Golgi is a highly dynamic organelle which undergoes continuous growth (through anterograde transport) and reconsumption (through retrograde transport) by the ER. Anterograde transport proceeds from ER exit sites to the pericentrosomal region by dynein-mediated transport of Golgi membranes on microtubules (Cole *et al*., 1996; Presley *et al*., 1997). It is possible that SCVs migrating from the cell periphery towards the MTOC are blocked by the Golgi apparatus, and that over time bacterial cell division leads to the formation of a microcolony that in turn gradually blocks anterograde traffic of Golgi membranes, giving rise to the frequently observed crescent- and doughnut-shaped Golgi in infected cells (Salcedo and Holden, 2003).

The SPI-2 T3SS and its effector SseG have an important role in the SCV–Golgi association phenotype, because vacuoles containing corresponding mutant strains are mainly scattered throughout the cell at this time-point (Salcedo and Holden, 2003). This raised the question whether vacuoles containing SPI-2 mutant bacteria fail to undergo centripetal migration following invasion, or if this occurs normally, but is followed by centrifugal movements, resulting in their scattered distribution in the cell. We found that vacuoles containing mutant bacteria localized to the MTOC/Golgi region with similar kinetics to those containing wt bacteria, but, if the bacteria lacked SseG, the SPI-2 T3SS, or an intact Golgi apparatus, vacuoles subsequently relocalized throughout the cell, resulting in a scattered phenotype. These observations are in agreement with previous results indicating that SPI-2 T3SS is required for SCV maintenance in a perinuclear location (Salcedo and Holden, 2003; Abrahams *et al*., 2006).

Live microscopy of infected epithelial cells revealed that the motility of SCVs changes during the course of infection. Following their initial slow saltatory migration towards the MTOC, the majority of vacuoles containing wt bacteria in the MTOC/Golgi region became relatively immobile. In contrast, between 6 and 8 h p.i. the majority of vacuoles containing SPI-2 mutant bacteria and a small proportion of wt SCVs were much more motile (displacing as much as 10 μm in 20 min), and moved erratically at higher speeds in a MT-dependent manner throughout the cell. Evidently, the lack of SseG or a functional SPI-2 T3SS results in an imbalance of motor activity on SCVs, causing their erratic and rapid movement in the cell. The speeds of these SCVs (0.06–0.09 μm s^−1^) is nevertheless much slower than those of bacterial pathogens which escape their vacuoles and polymerize actin at one pole of their cell surfaces to move within the cytoplasm. Actin-based motility of *Listeria*, *Shigella, Rickettsia, Burkholderia* and *Mycobacterium* generates speeds of up to 1.45 μm s^−1^ depending on pathogen and cell type, and subcellular location of the bacterium (Lacayo and Theriot, 2004; Stevens *et al*., 2006).

Interestingly, the speed of motile vacuoles containing wt but not mutant bacteria was correlated with their proximity to the Golgi, moving at higher speeds when they were further away from this organelle. This provides evidence that SseG acts to restrict the speed of SCVs that are located close to the Golgi, and suggests a tethering function for SseG in this region of the cell. Because we also show that Golgi disruption with BFA affects SCV positioning with similar kinetics to that of a SPI-2 mutant, it is possible that SseG might tether SCVs directly to Golgi membranes or associated molecules. Alternatively, SseG might be directly involved in microtubule motor recruitment, and persistent localization of SCVs in this region of the cell could be achieved through continuous dynein-based activity (Abrahams *et al*., 2006).

The localization of vacuoles containing wt bacteria to the Golgi or MTOC region clearly requires factors other than SseG. One of these is another SPI-2 T3SS effector, SseF (a membrane spanning protein with 30% amino acid identity to SseG). *sseF* mutants have an identical phenotype to *sseG* mutants (Abrahams *et al*., 2006; Deiwick *et al*., 2006) and both genetic and biochemical evidence exists for a functional interaction between these proteins (Deiwick *et al*., 2006). Both proteins localize mainly to the SCV membrane and cytoplasmic domains have been identified that are necessary for Golgi localization of SCVs (Salcedo and Holden, 2003; Abrahams *et al*., 2006). These proteins are likely to form a complex that interacts with host molecules to mediate tethering directly or indirectly through the control of microtubule motors. However, because the molecular mechanism of these two proteins remains unknown, whether or not SseG and SseF play a direct role in preventing movement remains to be elucidated. Although SseG and SseF exert a strong influence on SCV motility, the impact of the absence of these proteins is neither cumulative nor as pronounced as that observed with the SPI-2 null mutant. This indicates that other bacterial effectors also contribute to the control of SCV motility in an SseG/SseF-independent manner.

Another major question that remains to be answered concerns the physiological significance of the localization of SCVs in the MTOC/Golgi region. *C. trachomatis*, *Brucella abortus* and *Legionella pneumophila* all intercept membrane traffic at different stages of the secretory pathway (Hackstadt *et al*., 1996; Kagan and Roy, 2002; Carabeo *et al*., 2003; Celli *et al*., 2005), and one possibility is that fusion between SCVs and Golgi vesicular traffic might provide a source of nutrients for bacterial replication. However, although SCVs are often found in close apposition to Golgi membranes, and bacteria require an intact Golgi for replication, no evidence currently exists for the interception of vesicular traffic from the Golgi to SCVs (Salcedo and Holden, 2003; Kuhle *et al*., 2006).

## Experimental procedures

### Bacterial strains and growth conditions

*Salmonella enterica* serovar Typhimurium wt strain NCTC 12023 (Wray and Sojka, 1978) and its isogenic mutant derivatives *ssaV::aphT* (*ssaV*); (Deiwick *et al*., 1998) *sseG::aphT* (*sseG*)*; sseF::aphT* (*sseF*) (Hensel *et al*., 1998) and *sseFG::aphT* (*sseFG*) (Deiwick *et al*., 2006) were used in this study. The *sseG* and *sseF* mutants have been shown before to be non-polar (Hensel *et al*., 1998). Bacteria were grown in Luria–Bertani medium supplemented with ampicillin (50 μg ml^−1^) or kanamycin (50 μg ml^−1^) where appropriate.

### Cell culture and bacterial infection

HeLa (clone HtTA1) cell line was obtained from the European Collection of Cell Cultures (ECACC). Cells were maintained in Dulbecco's modified Eagle's medium (DMEM) containing glutamax (Invitrogen) and supplemented with 10% foetal calf serum (FCS; Sigma) at 37°C in a humidified atmosphere of 5% CO_2_ in air.

For bacterial infection, HeLa cells were seeded at a density of approximately 5 × 10^4^ cells per well in 24-well microtiter plates 16–20 h before infection. The cells were infected with *S.* Typhimurium strains grown to late-logarithmic phase, as described (Beuzon *et al*., 2000). In order to follow a synchronized population of bacteria, host cells were washed after 15 min of exposure to *S.* Typhimurium and subsequently incubated for 1 h in DMEM with 10% (v/v) FCS containing gentamicin (100 μg ml^−1^), to kill extracellular bacteria. The concentration of gentamicin in the medium was kept at 16 μg ml^−1^ for the rest of the experiment.

### Antibodies and reagents

The goat polyclonal anti-*Salmonella* antibody CSA-1 was purchased from Kirkegaard and Perry Laboratories (Gaithesburg, MD) and was used at a dilution of 1:1000. The rabbit anti-giantin antibody (Berkeley Antibody Company) was used at a dilution of 1:1000. Mouse monoclonal anti-γ-tubulin antibody (Sigma; clone GTU-88), was used at a dilution of 1:800. The rat anti-α-tubulin antibody (clone YL1/2 MCAP77G) was purchased from Serotec (Oxford, UK) and was used at a dilution of 1:200. Bound antibodies were detected using Cyanine 2 (Cy2)-, Cyanine 5 (Cy5-) and rhodamine red-X (RRX)-conjugated donkey anti-mouse, anti-rabbit and anti-goat antibodies (Jackson Immunoresearch Laboratories) and used at a dilution of 1:200.

Brefeldin A and nocodazole were obtained from Sigma. Stock solutions were made in dimethyl sulphoxide (DMSO) and kept at −20°C. Drugs were added directly to the culture medium. Working concentrations were 5 μg ml^−1^ for both BFA and nocodazole. To enable microtubule depolymerization cells were incubated for 20 min at 4°C to depolymerize microtubules, and then returned to 37°C in the presence of nocodazole (Cole *et al*., 1996). Under these conditions microtubules were depolymerized within 30 min, as detected with an anti-α-tubulin antibody (data not shown). Preliminary experiments confirmed that nocodazole treatment does not affect *S.* Typhimurium invasion (data not shown). To determine whether microtubules are involved in the migration of SCVs to the Golgi region, cells were incubated with nocodazole for 1 h prior to infection. For determination of microtubule-dependent movement after initial migration of SCVs, cells were treated with nocodazole at 2 h p.i. BFA was added to cell cultures 15 min after addition of bacteria.

### Immunofluorescence microscopy

For immunofluorescence microscopy, cell monolayers were fixed in methanol at −20°C for 5 min. Cells were then washed three times with phosphate-buffered saline, and labelled for immunofluorescence microscopy as previously described (Beuzon *et al*., 2000). Cell preparations were examined by confocal laser scanning microscopy (LSM510, Zeiss). Determination of distances of SCVs from the MTOC (γ-tubulin labelling) was achieved by scanning individual cells from apical to basal surface, identifying the location of the MTOC and then determining the distance of each SCV from the γ-tubulin labelling in the *x*/y plane as being either within or greater than 5 μm from the MTOC. Labelling of the MTOC using the anti-γ-tubulin antibody required methanol fixation; this leads to the collapse of apical membranes, resulting in flattened cell preparations and some loss of three-dimensional structure. Consequently, determination of bacterial distance from the MTOC could only be achieved in the *x*/y plane. Each infection was performed in triplicate and more than 50 infected cells were analysed for each experiment.

### Live-cell imaging

HeLa cells transfected with a vector encoding Mannosidase II-pEGFP-N1 (pMannII-EGFP) were infected with wt *sseG*, *sseF*, *sseFG* or *ssaV* mutant strains of *S.* Typhimurium carrying the pDsRed plasmid. This plasmid expresses a fluorescent-optmized DsRed protein under the control of the arabinose-inducible *P*_BAD_ promoter (Sorensen *et al*., 2003); MannII is a Golgi resident protein, mainly localized at the medial portion of the stack (Novikoff *et al*., 1983). Labelling of MannII-EGFP-expressing HeLa cells with anti-γ-tubulin and anti-giantin antibodies confirmed the close proximity of these markers (data not shown).

For live-cell imaging experiments, HeLa cells were seeded at a density of approximately 8 × 10^4^ cells per dish onto 35 mm glass-bottom culture dishes (MatTek, Ashland, MA), 16–20 h before transfection. The cells were then transfected for 20 h with pMannII-EGFP by using the jetPEI reagent (Qbiogene, USA), following manufacturer's instructions. Cells were infected with *S.* Typhimurium strains as described above. Before imaging, cells were washed and then incubated in imaging medium (DMEM without phenol red, containing 10% FCS, 110 μg ml^−1^ sodium pyruvate, 25 mM HEPES, 10 mM glutamine, 0.2% arabinose and 20 μg ml^−1^ gentamicin). Culture dishes were then sealed with parafilm (Pechiney, Menasha, WI, USA) and transferred to the stage of a Zeiss Axiovert 200 M microscope (Carl Zeiss, AG, Germany) located in a XL-2 temperature controlled incubator at 37°C. Samples were illuminated through a neutral density filter (Chroma Technology) and imaged with an ×63 oil objective. Images were captured sequentially with an ORCA AG Digital CCD Camera (Hamamatsu Photonics) using external filter wheels to switch between excitation/emission combinations of 480/525 for GFP and 565/620 for DsRed (Chroma Technology). Volocity software (Improvision) was used for both recording and image processing.

### Analysis of SCV motility

For qualitative analysis of SCV motility, an imaging frequency of one frame per min for 20–60 min was used. For quantitative analysis of SCV movement, an imaging frequency of 12 frames per min for up to 5 min was used. In each frame of the time-lapse sequences the *x*/*y* positions of the SCV and Golgi were estimated by identifying the centroid of the DsRed and MannII-EGFP fluorescence using Volocity software (Improvision).

To classify SCVs as undergoing large or small displacements, the difference between the maximum SCV displacement from the initial starting position was determined for each SCV trajectory. Values greater than 2 μm were classified as large displacements, while those with a value less than 2 μm were considered to be small displacements. SCV speeds for a single trajectory were calculated by dividing the total distance that the SCV travelled divided by the time of analysis. The directionality of SCV movement was evaluated essentially as described by Lacayo and Theriot (2004). Briefly, we evaluated directionality by plotting the *xy* co-ordinates of each SCV with all trajectories reorientated to start at *x,y* = 0 and the first displacement vector pointing in the same direction (+x, +y). To quantify the directionality of SCV movement, the cosine of the internal angle between displacement vectors was calculated and the values were averaged for each group of SCVs.

### Statistical analysis

All bar-graphs show the mean ± the standard error of the mean for three independent experiments. A two-tailed unpaired student's *t*-test was used to analyse all of the data sets apart from the live-imaging analysis of the displacement of SCVs over 20 min (Fig. 6) and the live imaging analysis of SCV speed verses distance from the Golgi. In these cases, a Fishers exact test and the Spearman correlation test were used respectively. Probabilities (p) values of 0.05 or less were considered significant. All statistical analysis was performed using Prism 4 software (GraphPad Software, San Diego, CA).

## Supplementary material

The following supplementary material is available for this article online:

Fig. S1 Live cell imaging analysis of sseF and sseFG mutant SCVs. Percentage of small (less than 2 mm) and large (greater than 2 mm) displacing sseF and sseFG SCVs between 4 h and 6 h p.i., and between 6 h and 8 h p.i.; number of SCVs in each category is indicated above the bar, as is the P-value from comparing the sseF and sseFG mutant SCVs with wt SCVs within the same time period. Images were acquired every min for 20 min.

Movie S1 Live cell imaging of DsRed wt S. Typhimurium and MannII-EGFP. Supplement to Fig. 2B. HeLa cells expressing MannII-EGFP were infected with DsRed expressing wt S. Typhimurium and imaged every min for 1 h. Two bacteria move slowly towards the MannII-EGFP marker. Movie is 300 times accelerated relative to real time.

Movie S2 Live cell imaging of DsRed wt S. Typhimurium and MannII-EGFP at 6 h 14 min. Supplement to Fig. 6C. HeLa cells expressing MannII-EGFP were infected with DsRed expressing wt S. Typhimurium and imaged every min for 20 min. Movie is 180 times accelerated relative to real time. Wt SCVs are seen in close proximity to the ManII-EGFP label and show restricted movement.

Movie S3 Live cell imaging of DsRed sseG mutant S. Typhimurium and MannII-EGFP at 7 h 27 min. Supplement to Fig. 6C. HeLa cells expressing MannII-EGFP were infected with DsRed expressing sseG S. Typhimurium and imaged every min for 20 min. Movie is 180 times accelerated relative to real time. Some SCVs are observed in close proximity to the ManII-EGFP marker and show little movement, and several SCVs move dramatically in and out of the focal plane.

Movie S4 Live cell imaging of DsRed ssaV mutant S. Typhimurium and MannII-EGFP at 7 h 32 min. Supplement to Fig. 6C. HeLa cells expressing MannII-EGFP were infected with DsRed expressing ssaV S. Typhimurium and imaged every min for 20 min. Movie is 180 times accelerated relative to real time. The majority of ssaV SCVs are observed to move erratically around the cell; no ssaV SCVs are in close proximity to the ManII-EGFP marker over the entire imaging time.

This material is available as part of the online article from: http://www.blackwell-synergy.com/doi/abs/10.1111/j.1462-5822. 2007.00977.x
