# Supplementary figures and images for: The SPI-2 type III secretion system restricts motility of Salmonella-containing vacuoles
Source: Cell Microbiol. 2007 Jun 7;9(10):2517–29. doi: 10.1111/j.1462-5822.2007.00977.x (PMC2062534; doi:10.1111/j.1462-5822.2007.00977.x)

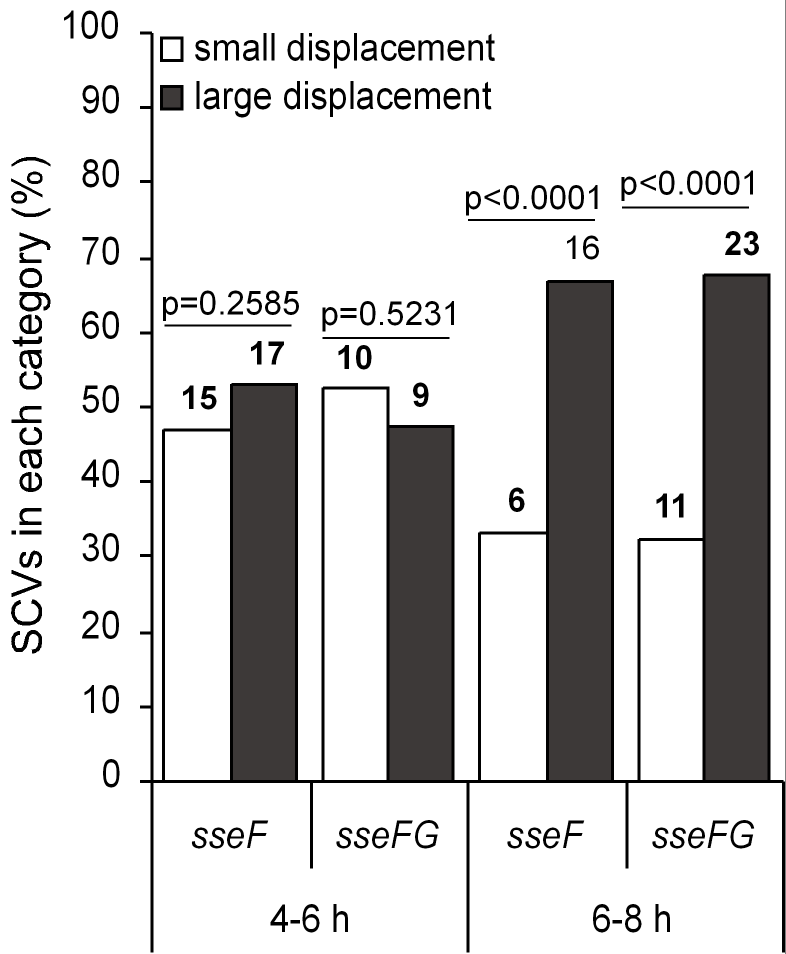

Supplement: Fig. S1 — Live cell imaging analysis of sseF and sseFG mutant SCVs. Percentage of small (less than 2 mm) and large (greater than 2 mm) displacing sseF and sseFG SCVs between 4 h and 6 h p.i., and between 6 h and 8 h p.i.; number of SCVs in each category is indicated above the bar, as is the P-value from comparing the sseF and sseFG mutant SCVs with wt SCVs within the same time period. Images were acquired every min for 20 min. [file cmi0009-2517-SD1.tif]
